# ﻿Three new species in *Tetrastemma* Ehrenberg, 1828 (Nemertea, Monostilifera) from sublittoral to upper bathyal zones of the northwestern Pacific

**DOI:** 10.3897/zookeys.1146.95004

**Published:** 2023-02-07

**Authors:** Natsumi Hookabe, Hisanori Kohtsuka, Yoshihiro Fujiwara, Shinji Tsuchida, Rei Ueshima

**Affiliations:** 1 School of Science, The University of Tokyo, 113-0033, Bunkyo, Japan The University of Tokyo Bunkyo Japan; 2 Misaki Marine Biological Station (MMBS), School of Science, The University of Tokyo, 238-0225, Miura, Japan The University of Tokyo Miura Japan; 3 Research Institute for Global Change (RIGC), Japan Agency for Marine-Earth Science and Technology (JAMSTEC), Yokosuka, 237-0061, Kanagawa, Japan Research Institute for Global Change Yokosuka Japan

**Keywords:** Deep sea, Eumonostilifera, Japan, marine invertebrate, Monostilifera, Nemertea, Pacific, Tetrastemmatidae

## Abstract

Monostiliferous nemerteans in the genus *Tetrastemma* Ehrenberg, 1828 are generally characterized as having four eyes, and they occur worldwide, from the intertidal zone to the deep-sea bottom. Recent extensive sampling of *Tetrastemma* has explored the high species diversity, including many undescribed forms, but phylogenic analysis has revealed non-monophyly of the genus. We herein describe three new species of the genus (*T.album***sp. nov.**, *T.persona***sp. nov.**, and *T.shohoense***sp. nov.**) from northwestern Pacific waters based on specimens collected by dredging or by use of a remotely operated vehicle at depths of 116–455 m. Since anatomical and histological characters traditionally used in systematics of the genus are sometimes interspecifically uniform, a histology-free approach is applied for the species descriptions in this study. To confirm the generic affiliation of the new species, a molecular phylogenetic analysis based on partial sequences of cytochrome *c* oxidase subunit I, 16S rRNA, 18S rRNA, 28S rRNA, and histone H3 genes was performed. Our result shows that all three new species are nested in a subclade formed by species from the North Pacific and American Atlantic, inferring that geographic distribution does not reflect the cladogenesis of *Tetrastemma*. Furthermore, two *Tetrastemma* species with a cylindrical stylet basis, *T.freyae*Chernyshev et al., 2020 from off the coast of India and Hawaii and *T.shohoense***sp. nov.** from Shoho Seamount, Japan, constitute a clade in the resulting tree.

## ﻿Introduction

A histology-free description with DNA barcoding has been progressively introduced to nemertean systematics in the past decade (e.g., Kajihara 2015; Gonzalez-Cueto et al. 2017; Simpson et al. 2017; Kajihara et al. 2018, 2022; Chernyshev et al. 2020; Hookabe et al. 2021a, b; Leiva et al. 2021; Abato et al. 2022). This approach has been applied to two cases, one of which is a description of species with internal characters interspecifically differentiated and observable without histology (e.g., number of proboscis branches in *Gorgonorhynchus* Dakin & Fordham, 1931 [Kajihara 2015; Hookabe et al. 2021a)]. In the other case, especially when internal morphology is uniform between most species in a genus, a species description has been performed solely based on characters examined *in-vivo* (shape of head, body coloration and markings, number of eyes, blood color, and stylet apparatus) [e.g., *Baseodiscus* Diesing, 1850 (Kajihara et al. 2022) and *Ototyphlonemertes* Diesing, 1863 (Kajihara et al. 2018)]. Recent descriptions of species in the genus *Tetrastemma* Ehrenberg, 1828, fitting the latter case, have been performed based on characters of living specimens without histological observations (Chernyshev et al. 2020; Hookabe et al. 2021b; Abato et al. 2022).

*Tetrastemma* is a species-rich genus in Monostilifera (Kajihara 2021), currently encompassing about 110 species from tropical to polar areas (Chernyshev et al. 2021). As the generic name suggests–a composite of the Latin feminine “*tetra*” (= four) + “*stemma*” (= simple eyes)–members in the genus are generally characterized by four eyes, but this feature is also found in other genera. Several species in *Tetrastemma* were described based on internal morphology; however, the internal characters were inferred to be almost homogenous within the genus by taxonomic reappraisal based on molecular phylogeny (Chernyshev et al. 2021). Recent examples of a histology-free approach based on characteristics studied *in-vivo* and molecular data are descriptions of *T.freyae*Chernyshev et al., 2020, *T.cupido* Hookabe, Kohtsuka & Kajihara, 2021, and *T.parallelos* Abato, Yoshida & Kajihara, 2022.

Here, we establish three new species based on specimens collected in 2019–2021 from the lower sublittoral to upper bathyal zones of Sagami Bay and the Nishi-Shichito Ridge. The descriptions are histology-free, based on characters of living specimens examined with a light microscope. To test phylogenetic relationships with the congeners, we performed molecular phylogenetic reconstruction using partial sequences of the 16S rRNA (16S), cytochrome *c* oxidase subunit I (COI), 18S rRNA (18S), 28S rRNA (28S), and histone H3 genes (H3).

## ﻿Materials and methods

Specimens were collected in 2019–2021 by use of a biological dredge in Sagami Bay (116–200 m) or a remotely operated vehicle (ROV) on Shoho Seamount of the Nishi-Shichito Ridge (455 m), northwestern Pacific Ocean. External morphology of the living specimens was documented on the vessel or in the laboratory with a Nikon D5600 digital SLR camera equipped with an AF-S DX Micro-NIKKOR 40mm f/2.8G macro lens (Nikon, Japan). A single specimen collected from Shoho Seamount was further observed under a compound light microscope by preparing a squeezed specimen with a cover slip and a glass slide. Specimens were anaesthetized with a few drops of bitterns Tenpi Nigari (Amashio, Japan); after the worms were relaxed, the posterior tips were preserved in 99% ethanol for DNA extraction and the rest of the body was fixed in Bouin’s fluid for 24–48 hours and later transferred to 70% ethanol. Type specimens have been deposited in the National Museum of Nature and Science, Tsukuba (**NSMT**), Japan.

DNA extraction, PCR amplification, and sequencing followed Hookabe et al. (2022). DNA sequences determined in the present study have been deposited in DDBJ/EMBL/GenBank (Table 1).

**Table 1.** List of species included in the phylogenetic analysis and DDBJ/EMBL/GenBank accession numbers for each gene. Country names of each species sampling location are abbreviated as follows: CA = Canada, JP = Japan, RU = Russia, USA = United States of America, and VE = Venezuela.

Species	Sampling location	16S	COI	18S	28S	H3	Source
*Tetrastemma* ‘*aequicolor*’ 24 QuI	Erineyskaya Inlet, RU	MZ231141	MZ216528	MZ231206	MZ231296	MZ216598*	Chernyshev et al. (2021)
*Tetrastemma* ‘*aequicolor*’ 25 QuI	Erineyskaya Inlet, RU	MZ231142	MZ216529	MZ231207	MZ231297	MZ216599*	Chernyshev et al. (2021)
*Tetrastemma* ‘*aequicolor*’ 26 QuI	Erineyskaya Inlet, RU	MZ231143	MZ216530	MZ231208	MZ231298	MZ216600*	Chernyshev et al. (2021)
*Tetrastemmaalbum* sp. nov.	Sagami Bay, JP	OQ248525	OQ249697	OQ248517	OQ248520	OQ248166	Present study
*Tetrastemmacupido*	Sagami Bay, JP	OK428649	OK414013	OK428689	OK428648	–	Hookabe et al. (2021b)
*Tetrastemmanigrifrons*	CA	MZ231144	MZ216531	MZ231209	MZ231299	MZ216601	Chernyshev et al. (2021)
	Oregon, USA	MZ231145	MZ216532	MZ231210	MZ231300	MZ216602	Chernyshev et al. (2021)
	California, USA	MZ231146	MZ216533	MZ231211	MZ231301	MZ216603	Chernyshev et al. (2021)
*Tetrastemmastimpsoni*	JP	MZ231147	MZ216534	MZ231212	MZ231301	MZ216604	Chernyshev et al. (2021)
	RU	MZ231148	MZ216535	MZ231213	MZ231303	MZ216605	Chernyshev et al. (2021)
	Iturup, RU	MZ231149	MZ216536	MZ231214	MZ231304	MZ216606	Chernyshev et al. (2021)
*Tetrastemmaelegans* B2	York River, USA	MZ231156	MZ216543	MZ231222	MZ231312	MZ216614	Chernyshev et al. (2021)
*Tetrastemmaelegans* C2	USA	MZ231157	MZ216544	MZ231223	MZ231313	MZ216615	Chernyshev et al. (2021)
*Tetrastemmaelegans* D2	York River, USA	MZ231158	–	MZ231224	MZ231314	–	Chernyshev et al. (2021)
*Tetrastemmaenteroplecta* A6	Florida, USA	MZ231159	MZ216546	MZ231225	MZ231314	MZ216616	Chernyshev et al. (2021)
*Tetrastemmaenteroplecta* E3	Florida, USA	MZ231160	–	MZ231226	MZ231316	MZ216618	Chernyshev et al. (2021)
*Tetrastemmaenteroplecta* B7	VE	MZ231161	–	MZ231227	MZ231317	–	Chernyshev et al. (2021)
*Tetrastemmafreyae*	Hawaii, USA	–	MT247877	MZ231229	MZ231319	MT247879	Chernyshev et al. (2020)
*Tetrastemmamerulum* F2	Florida, USA	MZ231163	MZ216550	MZ231231	MZ231321	MZ216622	Chernyshev et al. (2021)
*Tetrastemmamerulum* H5	Florida, USA	MZ231164	MZ216551	MZ231232	MZ231322	MZ216623	Chernyshev et al. (2021)
*Tetrastemmapersona* sp. nov.	Sagami Bay, JP	OQ248526	OQ249698	OQ248518	OQ248521	OQ248167	Present study
*Tetrastemmareticulatum*	California, USA	MZ231168	MZ216556	MZ231238	MZ231328	MZ216629	Chernyshev et al. (2021)
*Tetrastemmashohoense* sp. nov.	Shoho Seamount, JP	OQ248524	OQ249700	–	–	–	Present study
*Tetrastemma* sp. F7	Florida, USA	MZ231173	MZ216564	MZ231246	MZ231336	MZ216637	Chernyshev et al. (2021)
*Tetrastemma* sp. GM 1	Gulf of Mexico, USA	MZ231175	–	MZ231248	MZ231338	MZ216639	Chernyshev et al. (2021)
*Tetrastemma* sp. GM 2	Florida, USA	MZ231176	MZ216565	MZ231249	MZ231339	MZ216640	Chernyshev et al. (2021)
*Tetrastemma* sp. GM 3	Gulf of Mexico, USA	MZ231177	–	MZ231250	MZ231340	MZ216641	Chernyshev et al. (2021)
*Tetrastemma* sp. I	Iturup, RU	MZ231179	–	MZ231252	MZ231342	MZ216643	Chernyshev et al. (2021)
*Tetrastemma* sp. IP	Iturup, RU	MZ231180	MZ216567	MZ231253	MZ231343	MZ216644	Chernyshev et al. (2021)
*Tetrastemma* sp. J 1TjS	Simushir, RU	MZ231182	MZ216570	MZ231256	MZ231346	MZ216647	Chernyshev et al. (2021)
*Tetrastemma* sp. J 3TjS	Simushir, RU	MZ231183	MZ216571	MZ231257	MZ231347	MZ216648	Chernyshev et al. (2021)
*Tetrastemma* sp. J 4TjS	Simushir, RU	MZ231184	MZ216572	MZ231258	MZ231348	MZ216649	Chernyshev et al. (2021)
*Tetrastemma* sp. M1	Urup, RU	–	MZ216573	MZ231259	MZ231349	MZ216650	Chernyshev et al. (2021)
*Tetrastemma* sp. M2	Urup, RU	–	MZ216574	MZ231260	MZ231350	MZ216651	Chernyshev et al. (2021)
*Tetrastemma* sp. Ofunato	Off Ofunato, JP	OQ248527	OQ249699	OQ248519	OQ248522	OQ248168	Chernyshev et al. (2021)
*Tetrastemma* sp. S 1TsS	Simushir, RU	–	MZ216575	MZ231261	MZ231351	MZ216652	Chernyshev et al. (2021)
*Tetrastemma* sp. S 2TsS	Simushir, RU	–	MZ216576	MZ231262	MZ231352	MZ216653	Chernyshev et al. (2021)
*Tetrastemma* sp. U 13TsU	Urup, RU	MZ231185	MZ216577	MZ231263	MZ231353	MZ216654	Chernyshev et al. (2021)
*Tetrastemma* sp. U 18TsU	Urup, RU	MZ231186	MZ216578	MZ231264	MZ231354	MZ216655	Chernyshev et al. (2021)
*Tetrastemma* sp. UR	Urup, RU	MZ231187	–	MZ231265	MZ231355	MZ216656	Chernyshev et al. (2021)

*Erroneously registered in GenBank under the taxon name *Quasitetrastemmanigrifrons*.

To elucidate phylogenetic positions of specimens examined, we performed phylogenetic analyses based on the maximum-likelihood (ML) method. The newly obtained sequences from four *Tetrastemma* species were aligned using MAFFT v. 7 (Katoh and Standley 2013) employing L-INS-i strategy with sequences of other species in the genus, most of which were recently determined by Chernyshev et al. (2021). Ambiguous nucleotide sites in the dataset were removed with Gblocks v. 0.91b (Castresana 2000) using a less stringent option, resulting in 380-bp 16S, 626-bp COI, 1738-bp 18S, 505-bp 28S, and 329-bp H3. The ML analyses were performed with RAxML-NG (Kozlov et al. 2019), for which the best-fit partition scheme and substitution model were selected using PartitionFinder v. 2.1.1 (Lanfear et al. 2017). Nodal support values were derived from 1000 bootstrap pseudoreplicates.

## ﻿Result

### ﻿Systematics

**Genus *Tetrastemma* Ehrenberg, 1828**

#### ﻿ Tetrastemma album sp. nov.

Taxon classificationAnimaliaHoplonemerteaTetrastemmatidae

2E514ABC-244C-55E4-AFBD-77128ECF605E

https://zoobank.org/F73378DB-B867-4ABA-A2D7-18CF781D10A7

Fig. 2A–C [New Japanese name: misaki-oshiroi-himomushi]

##### Etymology.

The species name is derived from the Latin *album* (white), referring to pure white body of the new species. The Japanese name is named after the white powder foundation traditionally used by Maiko, Geisha, Kabuki actors in Japan.

##### Material examined.

***Holotype***: NMST-NE-H-06, unsectioned complete specimen except for the posterior tip, fixed in Bouin’s fluid and later preserved in 70% ethanol, posterior tip preserved in 99% ethanol, collected on March 12, 2021 by NH, biological dredge (R/V *Rinkai-maru*) at depths of 144–200 m, off Jogshima (35°07.41'N, 139°34.11'E–35°07.32'N, 139°33.572'E), Miura, Kanagawa, Japan, NW Pacific.

##### Description.

Head spatulate to rounded in profile (Fig. 2A–C), demarcated by posterior cephalic furrows from body (Fig. 2A). Before anesthetization, body of a live specimen 17 mm long and 1.0–1.2 mm wide. Body uniformly pale colored, without longitudinal or transverse stripe markings (Fig. 2A). Pure white transverse cephalic patch present between anterior and posterior pairs of eyes (Fig. 2B). Head not wider than maximum body width (Fig. 2A–C). A pair of cephalic furrows present; anterior pair not meeting mid-dorsally and ventrally curving anteriorly but not reaching to proboscis pore; posterior pair V-shaped and barely meeting mid-dorsally (Fig. 2B) and running transversely on ventral surface (Fig. 2C). Cerebral ganglia and blood not red and probably uncolored. Internal organs (proboscis, foregut, and intestine) visible as pale regions. Four reddish brown eyes regular in size (Fig. 2B).

##### Type locality and distribution.

**Figure 1.**
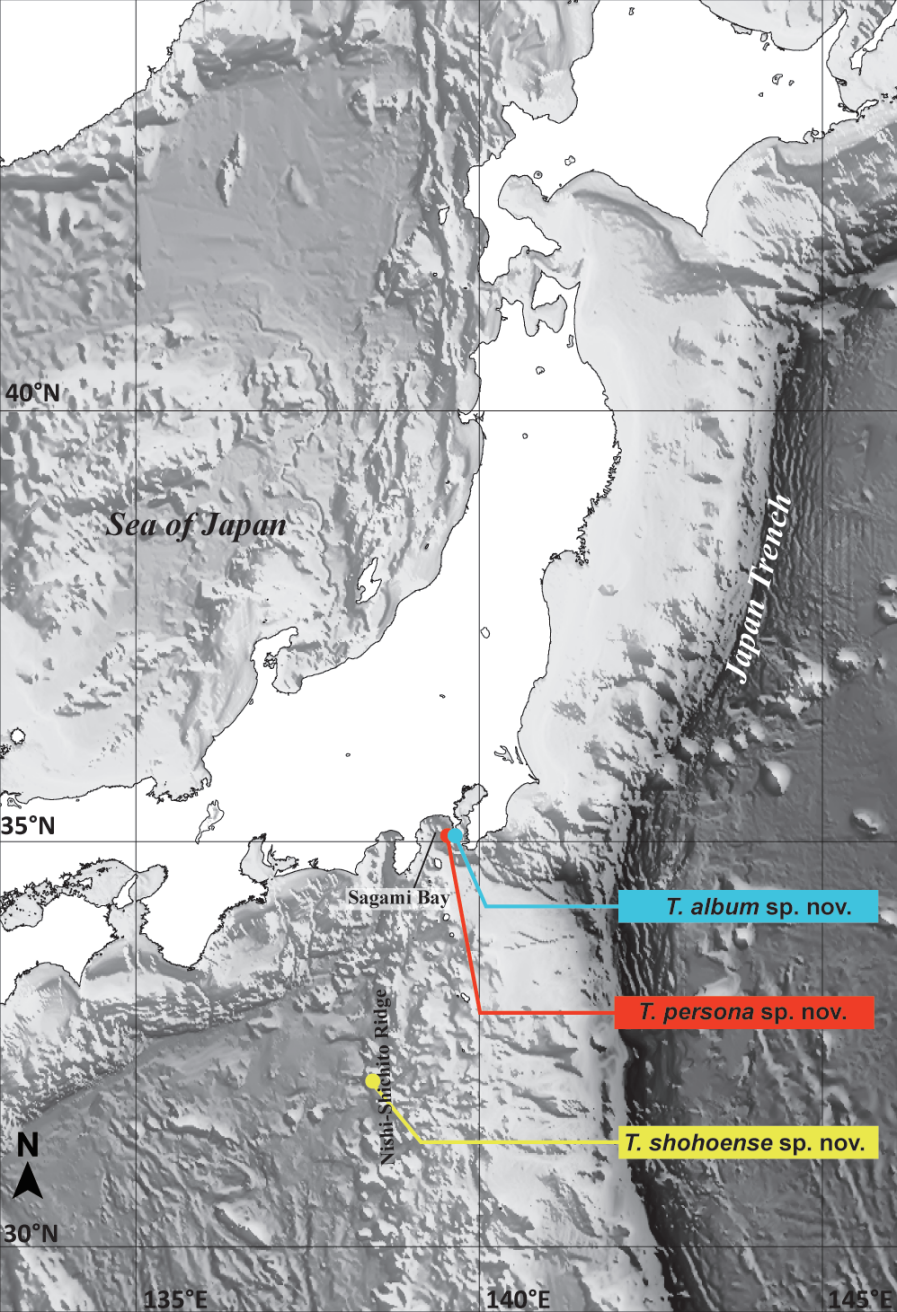
Collection sites of the specimens examined in the present study.

The species is only known from the type locality, Sagami Bay, Kanagawa Prefecture, Japan, at depths of 144–200 m (Fig. 1).

**Figure 2.**
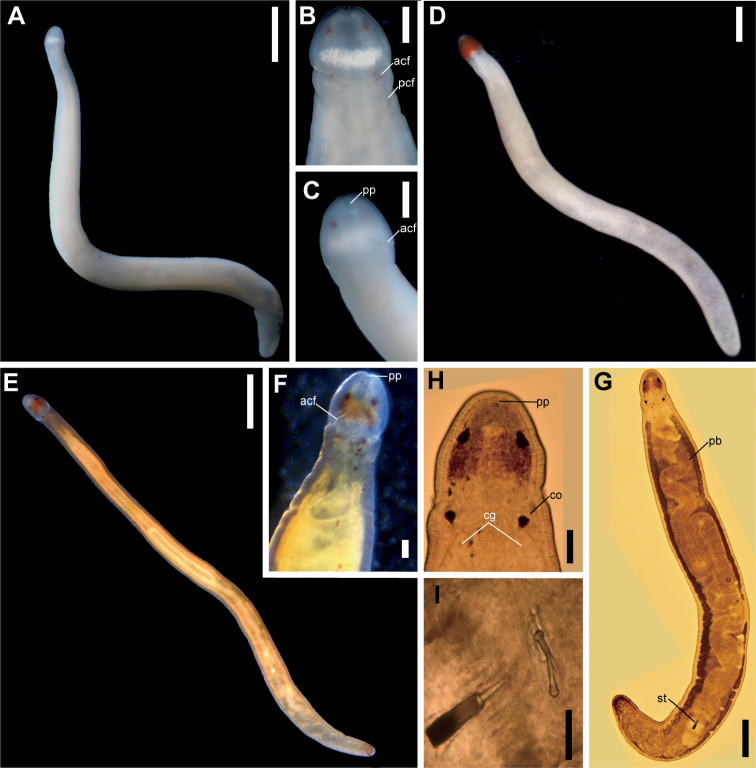
Holotype specimens of new *Tetrastemma* species; photographs were taken in life by NH **A–C***T.album* sp. nov. **A** complete body, dorsal view **B** head, dorsal view **C** head, ventral view **D***T.persona* sp. nov., complete body, dorsal view **E–I***T.shohoense* sp. nov. **E** complete body **F** head, ventral view **G** squeezed specimen under a cover slip, complete body, dorsal view **H** head, dorsal view **I** stylet apparatus. Abbreviations: acf, anterior cephalic furrow; pcf, posterior cephalic furrow, cg, cerebral ganglia; co, cerebral organ; pb, proboscis; pp, proboscis pore. Scale bars: 2 mm (**A**); 500 μm (**B, C, G**); 1 mm (**D, E**); 100 μm (**F, H**); 50 μm (**I**).

##### Remarks.

Having a pure white cephalic patch on a uniformly pale body, *T.album* sp. nov. differs from all the described species. *Tetrastemmacoronatum* (Quatrefages, 1846), *T.diadema* Hubrecht, 1879, *T.olgarum*Chernyshev 1998, and *T.pseudocoronatum*Chernyshev 1998 have white cephalic patches but are distinguished from *T.album* sp. nov. in possessing a light brown to dark transverse band on the head. *Tetrastemmaalbomaculatum* Chernyshev, 2016 also possesses a white cephalic patch but differs from the new species in having a pale-ochre body dorsally spotted with small white dots (Chernyshev 2016).

#### ﻿ Tetrastemma persona sp. nov.

Taxon classificationAnimaliaHoplonemerteaTetrastemmatidae

220BA17D-A947-5A01-96EB-33B4D973ABA5

https://zoobank.org/E3E48065-E551-477D-9041-89DEE011DEB0

Fig. 2D [New Japanese name: misaki-kamen-himomushi]

##### Etymology.

The species name is derived from the Latin *persōna* (mask), referring to a broad cephalic patch of the new species masking eyes and internal organs in head region. The Japanese name “kamen” means a mask in Japanese.

##### Material examined.

***Holotype***: NMST-NE-H-07, unsectioned complete specimen except for the posterior tip, fixed in Bouin’s fluid and later preserved in 70% ethanol, posterior tip preserved in 99% ethanol, collected on July 31 2020 by NH, biological dredge (R/V *Rinkai-maru*) at depths of 116–211 m, off Jogshima (35°08.32'N, 139°32.857'E–35°08.40'N, 139°32.504'E), Miura, Kanagawa, Japan, NW Pacific. ***Paratype***: NMST-NE-P-08, unsectioned complete specimen fixed in Bouin’s fluid and later preserved in 70% ethanol, collected on the same date and locality as the holotype.

##### Description.

Head slightly narrower than middle part of body and weakly demarcated from trunk (Fig. 2D). Before anesthetization, body of a live specimen 7.0–10 mm long and 0.8–1.0 mm wide. Body uniformly pale to yellow colored without longitudinal or transverse stripe markings (Fig. 2D). Vermilion-red cephalic patch spade-shaped (Fig. 2D), covering both anterior and posterior pairs of eyes (Fig. 2D) but not posteriorly reaching to anterior pair of cephalic furrows; eyes regular in sizes. A posterior pair of cephalic furrows not well distinguished probably due to the small body size. Cerebral ganglia and blood not red and probably uncolored. Internal organs (proboscis, foregut, and intestine) not well visible through body wall. Rhynchocoel visible as whitish region through body wall, extending about 1/2–2/3 of the body length.

##### Type locality and distribution.

The species is only known from the type locality, Sagami Bay, Kanagawa Prefecture, Japan, at depths of 116–211 m (Fig. 1, Table 1).

##### Remarks.

*Tetrastemmapersona* sp. nov. has atypically short rhynchocoel in the genus and most resembles *T.roseocephalum* (Yamaoka, 1947) and *T.yamaokai* Iwata, 1954 in having a pale body without any markings and a red cephalic patch. Pattern variation of a cephalic patch (shield shape or horse-shoe shape) was reported in both *T.roseocephalum* and *T.yamaokai*; referring to the original description of *T.yamaokai*, the name may be a junior synonym of *T.roseocephalum*, as suggested by Kajihara (2007). The external morphology of *T.persona* sp. nov. is similar to a form with a shield-shaped cephalic patch of *T.roseocephalum* (Iwata 1954).

**Figure 3.**
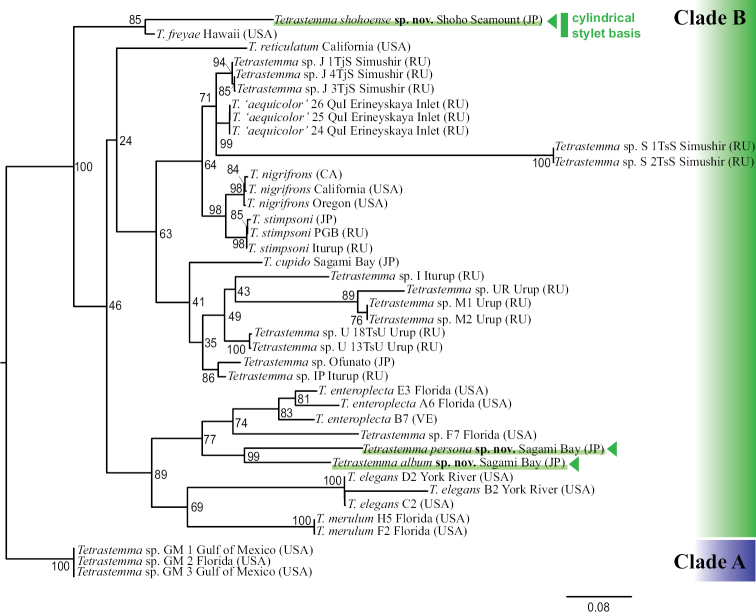
A maximum-likelihood (ML) tree based on concatenated sequences of two mitochondrial [16S rRNA (16S; 380 bp) and cytochrome *c* oxidase subunit I (COI; 626 bp)] and three nuclear gene markers [18S rRNA (18S; 1738 bp), 28S rRNA (28S; 505 bp), and histone H3 (H3; 329 bp)]. Numbers near each node are support values generated by a separate partitioned ML bootstrap analysis with 1000 replicates. Country names of each species sampling location are abbreviated as follows: CA = Canada, JP = Japan, RU = Russia, USA = United States of America, and VE = Venezuela.

The subtle difference in the shape of cephalic patch between *T.persona* sp. nov. (spade-shaped) and *T.roseocephalum* (shield-shaped) was supported by our molecular analysis. The new species did not constitute a clade with *T.roseocephalum* but with *T.album* sp. nov. (Fig. 3); *T.roseocephalum* belongs to Clade C of Chernyshev et al. (2021).

An uncorrected genetic distance based on 657 bp of COI was 16% between *T.album* sp. nov. and *T.persona* sp. nov., comparable with interspecific values observed among Monostilifera (e.g., Sundberg et al. 2016; Hookabe et al. 2022).

#### ﻿ Tetrastemma shohoense sp. nov.

Taxon classificationAnimaliaHoplonemerteaTetrastemmatidae

E64BFBDD-40A5-5912-9F6C-3659A7D127E0

https://zoobank.org/9D1CD900-900F-4114-8853-16C480FDD75D

Fig. 2E–I [New Japanese name: shoho-kakubari-himomushi]

##### Etymology.

The species is named after the type locality, Shoho Seamount of the Nishi-Shichito Ridge, Japan.

##### Material examined.

***Holotype***: NMST-Nem-H-05, unsectioned complete specimen except for the posterior tip, fixed in Bouin’s fluid, posterior tip preserved in 99% ethanol, collected on November 29 2020 by NH, by use of ROV*KM-ROV* (dive #123) during KM20-10C cruise of R/V *Kaimei*, at a depth of 455 m, near the summit of Shoho Seamount of the Nishi-Shichito Ridge (32°19.39'N, 138°44.48'E), Japan, NW Pacific.

##### Description.

Head spatulate in profile (Fig. 2E–H), not well demarcated from body by anterior cephalic furrows (Fig. 2E). Before anesthetization, body of a live specimen 5.5 mm long and 0.3 mm wide. Background body color generally white, tinged with bright yellow to orange, and almost transparent (Fig. 2E). Head with a red rectangle cephalic patch without extending behind a posterior pair of eyes (Fig. 2E, H). Anterior pair of cephalic furrows present (Fig. 2F) but posterior one not well distinguished. Cerebral ganglia and blood uncolored (Fig. 2G, H). Alimentary canals visible as bright yellow organs through body wall (Fig. 2E). Proboscis pale, extending about 3/4 of the body length (Fig. 2E). Four brown eyes present; anterior pair slightly larger than posterior ones (Fig. 2H).

Stylet basis cylindrical, 55.0 μm in length and 25.0 μm in maximum width; central stylet smooth, 47.0 μm in length; (stylet length) / (basis length) ratio 0.85 (Fig. 2I). Two accessory stylet pouches present, each containing two stylets (Fig. 2I).

##### Type locality and distribution.

The species is only known from the type locality, Shoho Seamount of the Nishi-Shichito Ridge, Japan, at a depth of 455 m (Fig. 1), among the sandy sediments on rocky substrates.

##### Remarks.

Having a dark cephalic patch and cylindrical stylet basis and lacking a longitudinal line on the dorsal surface of the body, *T.shohoense* sp. nov. resembles *T.freyae*Chernyshev et al., 2020 originally described based on Hawaiian and Indian specimens. The new species is differentiated from *T.freyae* in the color of the cephalic patch as well as the non-flared posterior margin of the cylindrical stylet basis.

A genetic distance based on COI between *T.shohoense* sp. nov. and *T.freyae* (specimens from Hawaii (MT247877) and India (MT247878) was 12.6%; the value is comparable with interspecific values observed among Monostilifera (e.g., Sundberg et al. 2016; Hookabe et al. 2022).

### ﻿Molecular phylogeny

The sequence data set for molecular phylogenetic analyses in the present study is primarily based on Chernyshev et al. (2021). Since we confirmed that our new species are nested in *Tetrastemma* Clade B of Chernyshev et al. (2021), we used three species in Clade A (*Tetrastemma* sp. GM1 Gulf of Mexico, *Tetrastemma* sp. GM2 USA FL, and *Tetrastemma* sp. GM3 Gulf of Mexico) as outgroup taxa (Fig. 3). Clade B was subdivided into two clades with a full support value, one of which was a clade formed by *T.freyae* and *T.shohoense* sp. nov. The two species are characterized by having a cylindrical stylet basis in the proboscis. In the other subclade in Clade B, *T.album* sp. nov. and *T.persona* sp. nov. were included (Fig. 3). A clade constituted by newly described species, *T.album* sp. nov. and *T.persona* sp. nov., from Sagami Bay (Japan) with 99% of BS, was nested in the American Atlantic clade formed by *T.elegans* (Girard, 1852) (Virginia), *T.enteroplecta* (Corrêa, 1954) (Florida and Venezuela), *T.merulum* (Corrêa, 1954) (Florida), and *Tetrastemma* sp. F7 (Florida). The clade formed by *T.album* sp. nov. and *T.persona* sp. nov. was sister-related to a clade formed by *T.enteroplecta* (Florida and Venezuela) and *Tetrastemma* sp. F7 (Florida) with 77% of BS (Fig. 3).

## ﻿Discussion

Three species herein described (*T.album* sp. nov., *T.persona* sp. nov., and *T.shohoense* sp. nov.) fell within a clade referred to as *Tetrastemma* Clade B of Chernyshev et al. (2021) (Fig. 3). One of the findings from the tree is that two species with cylindrical stylet basis, *T.freyae* and *T.shohoense* sp. nov., formed a clade regardless of the differences in habitat and collection depths of these two species; *T.freyae* was described based on specimens collected from live corals and mussel beds at depths shallower than 3 m in Hawaii and India (Chernyshev et al. 2020), while *T.shohoense* sp. nov. was found from sandy sediments in bathyal zone in Japan. A cylindrical stylet basis is likely to be acquired independently at least twice in Clade B (*T.freyae* and *T.shohoense* sp. nov.) and Clade C (*T.albomaculatum* and *T.parallelos*).

The other thing we can see on the phylogenetic tree is that *T.album* sp. nov. and *T.persona* sp. nov. are nested in a clade formed by several American Atlantic species, *T.enteroplecta*, *T.elegans*, *T.merulum*, and *Tetrastemma* sp. F7 (Chernyshev et al. 2021) (Fig. 3). A previous molecular analysis has inferred that *Tetrastema* clade B is subdivided into geographically distinct structures: North Pacific and American Atlantic subclades (Chernyshev et al. 2021). To obtain a more accurate picture of *Tetrastemma* phylogeny and speciation, again, further sampling of taxa, without bias toward shallow-water species, is needed for future phylogenetic analyses.

## Supplementary Material

XML Treatment for Tetrastemma album

XML Treatment for Tetrastemma persona

XML Treatment for Tetrastemma shohoense
